# Genome Sequence of *Paenibacillus wulumuqiensis* sp. nov., a Bioflocculant-Producing Species

**DOI:** 10.1128/genomeA.00795-15

**Published:** 2015-07-16

**Authors:** Qian Wu, Liying Zhu, Ling Jiang, Xian Xu, Qing Xu, Zhidong Zhang, He Huang

**Affiliations:** a College of Biotechnology and Pharmaceutical Engineering, Nanjing Tech University, Nanjing, People's Republic of China; b College of Sciences, Nanjing Tech University, Nanjing, People's Republic of China; c College of Food Science and Light, Industry, Nanjing Tech University, Nanjing, People's Republic of China; d Institute of Microbiology, Xinjiang Academy of Agricultural Sciences, Urumqi, Xinjiang, People's Republic of China

## Abstract

*Paenibacillus wulumuqiensis* sp. nov. is a novel strain that can produce bioflocculants. Here, we report 5.37-Mb assembly of its genome sequence and other useful information, including the coding sequences (CDSs) responsible for the biosynthesis of polysaccharide-based bioflocculants, cold-shock protein, and vitamin production.

## GENOME ANNOUNCEMENT

Over the past decades, more and more attention has been paid worldwide to the field of industrial wastewater treatment, and the use of bioflocculants has proved to be a superior method for removing pollutants from wastewater due to its harmlessness and biodegradability compared with many existing inorganic and organic high-polymer flocculants (1, 2). Among the bioflocculants, the polysaccharide-based bioflocculants (PSBs) have drawn particular attention in recent years, especially with respect to wastewater treatment, due to their unique structure, excellent physical-chemical and rheological properties, high efficiency, and excellent selectivity (3). Recently, our group isolated, from a cold spring sample from Xinjiang Uyghur Autonomous Region (China), a novel species of *Paenibacillus wulumuqiensis* sp. nov. (= CPCC 100602^T^ = JCM 30284^T^), which can produce PSBs naturally (4). Comparisons with 16S rRNA gene sequences revealed that the novel strain had the highest similarity to *P. hunanensis* FeL05^T^ (96%) (5). However, the phylogenetic distances from recognized species indicated that *P. wulumuqiensis* sp. nov. is not affiliated to any of these recognized species and the proportion of the saturated straight-chain fatty acid C_16:0_ was relatively high (4). We can therefore conclude that this strain represents a novel species of the genus *Paenibacillus*. As a result, studying the genetic information and characteristics of *P. wulumuqiensis* sp. nov. is desired to further investigate its mechanism.

Here, we present the draft genome sequence of strain *P. wulumuqiensis* sp. nov., obtained using the Illumina HiSeq 2000 system. The reads were assembled with SOAPdenovo (6, 7), and the sequence was annotated using the RAST annotation server (8); a KEGG metabolic pathway was also constructed. A library containing 500 inserts was constructed, and sequencing was performed based on the paired-end strategy of 484 reads to produce 773 Mb of filtered sequences, representing a 125-fold coverage of the genome. The sequence of *P. wulumuqiensis* sp. nov. comprises 5,240,711 bp with a G+C content of 49.19%, which was assembled into 41 contigs and 33 scaffolds. It contains 4,664 open reading frames (ORFs), 80 tRNA genes, and 3 rRNA genes identified by Glimmer version 3.02 (9), GeneMark (10), tRNAscan-SE (11), and RNAmmer (12).

According to the genomic analysis of strain *P. wulumuqiensis* sp. nov., we identified 52 ORFs related to the polysaccharide-based bioflocculants. Strain *P. wulumuqiensis* sp. nov. has 12 ORFs related to trehalose synthesis, which makes us believe that it could be related to a cold-resistance mechanism since trehalose is regarded as a molecular chaperone. Seven ORFs related to the cold-shock protein were also found in this novel strain. Additionally, the biosynthesis of vitamin B_1_ was annotated in *P. wulumuqiensis* sp. nov., as there were 55 ORFs related to thiamine metabolism. A complete genome sequence will be included in the future to reveal the unique molecular characteristics of strain *P. wulumuqiensis* sp. nov.

### Nucleotide sequence accession numbers. 

This whole-genome shotgun project has been deposited at DDBJ/EMBL/GenBank under the accession number LAQP00000000. The version described in this paper is the first version, LAQP01000000.
